# Activated innate lymphoid cell populations accumulate in human tumour tissues

**DOI:** 10.1186/s12885-018-4262-4

**Published:** 2018-03-27

**Authors:** Maryam Salimi, Ruozheng Wang, Xuan Yao, Xi Li, Xiyan Wang, Yuhui Hu, Xumei Chang, Peiwen Fan, Tao Dong, Graham Ogg

**Affiliations:** 1 MRC Human Immunology Unit, Weatherall Institute of Molecular Medicine, Radcliffe Department of Medicine, University of Oxford, Oxford, OX3 9DS UK; 2 Affiliated Tumor Hospital of Xinjiang Medical University, Ürümqi, China; 3 Key Laboratory of Cancer Immunity and Radiotherapy of Chinese Academy of Medical Sciences, Ürümqi, China; 4 Chinese Academy of Medical Sciences-Oxford Institute, Nuffield Department of Medicine, University of Oxford, Oxford, UK

**Keywords:** Innate lymphoid cells, Breast cancer, Gastrointestinal cancer, Immune checkpoint

## Abstract

**Background:**

Innate lymphoid cells (ILC) are part of a heterogeneous family of haematopoietic effector cells which lack re-arranged antigen-specific receptors. They promote host defense and contribute to tissue and metabolic homeostasis, wound healing and immune surveillance. Their role in human cancer immunity is less defined, and therefore we aimed to identify the frequency and phenotype of distinct ILC groups in various types of cancer.

**Methods:**

Tissue samples and peripheral blood were collected from patients undergoing surgical resection of gastrointestinal and breast tumours. Single cell suspension of tumour tissue was immediately obtained following surgery using tumour dissociation.

**Results:**

We observed significantly higher frequencies of ILC2 (*p* value: 0.04) in malignant breast cancer tissue and significantly higher frequencies of group 1 ILC (p value: 0.001) in malignant gastrointestinal tumours. Tumour infiltrating ILC were found to show an activated phenotype with higher expression of MHC-II, KLRG1, early activation marker CD69 and CD44.

**Conclusions:**

Activated innate lymphoid cells infiltrate tumours dependent on tumour type and location.

**Electronic supplementary material:**

The online version of this article (10.1186/s12885-018-4262-4) contains supplementary material, which is available to authorized users.

## Background

Significant advances have been made in understanding the immune surveillance mechanisms in identifying and destroying tumour cells [1, 2]. The importance of adaptive immunity [3] as a key effector cell mechanism in cancer control is now well documented. In colorectal cancer, infiltration of effector memory CD8+ T cells is associated with a reduction in early invasion and improved survival [4]. Over the past 20 years many tumour specific antigens have been identified which has led to clinical trials of vaccines, adjuvants, therapeutic antibodies and adoptive T cell immunotherapies. However key limitations remain in targeted cancer immunotherapy [5]. The prevailing ability of the tumour cells to evade recognition by the immune system gave rise to the concept of immunoediting [6].

Another cell population that has been implicated in immune defence against tumour cells are natural killer (NK) lymphocytes. Infiltration of NK cells is a recognised feature of most cancers [2, 7]. Unlike adaptive immune responses, NK cells are not antigen specific and rather identify tumour cells through expression of stress molecules or lack of major histocompatibility complex (MHC-I) receptors ‘missing self’ [8]. NK cells are a prototype of a newly discovered family of innate haematopoietic cells (innate lymphoid cells, ILC) with effector functions parallel to adaptive T cell lymphocytes. Three groups of ILC have been characterized based on signature cytokine secretion profile and expression of transcription factors. Group 1 ILC have cytotoxic abilities and the capacity to produce IFN-γ and TNF-α. Their effector function depends on expression of T-bet and Eomes transcription factors. NK cells and ILC1 contribute to this group of ILC [9]. Group 2 ILC are mainly implicated in allergic responses [10, 11] and defence against helminth infections but also have roles in epithelial repair and metabolic homeostasis. They produce IL-13, IL-4 and IL-5 and similar to Th2 lymphocytes express GATA-3 and ROR-α transcription factors [12]. Group 3 ILC are a RORγt-dependent group of ILC including lymphoid tissue inducers (LTi), natural cytotoxicity receptor (NCR)^+^ and NCR^−^ ILC3s. LTis are essential for generation of lymph nodes, Peyer’s patches and isolated lymphoid follicles. Similar to Th17 and Th22, ILC3s produce IL-17A and IL-22 [12, 13].

Given that ILC are mainly located in pathogen entry sites e.g. mucosal surfaces of gastrointestinal tract, respiratory system and the skin, their main function was considered to be protection against infections. However, recent data show that ILC also contribute to immunity against cancer in mice [14]. Dadi and colleagues have identified an unconventional population of TCR^−^ NK1.1^+^ CD49a^hi^ cells in mammary tumour tissue of MMTV-PyMT (PyMT) mice [15]. Due to their distinct features they were designated ‘type 1-like ILC’. These cells expressed high levels of granzyme B and exhibited cytolytic activity towards malignant cells [15]. In another study, IL-22 produced by ILC3s promoted bacteria-induced colorectal cancer (CRC) in genetically susceptible 129SvEv.RAG^−/−^ mice when infected with H.*hepaticus* and treated with the carcinogen azoxymethane (AOM) [16]. Furthermore, higher frequencies of CD3^−^ IL-22^+^ cells have been observed in the tumour tissue of patients with CRC [16].

ILC2 and ILC2 related genes (RORα, GATA3, T1/ST2, IL-17RB, CRTH2, IL-33, IL-5, and IL-4) were increased in the peripheral blood of patients with gastric cancer compared to healthy controls [17]. Jovanovic et al. showed accumulation of IL-13-producing Lin^−^ Sca-1^+^ ST2^+^ innate lymphoid cells (ILC) and reduction of NK cells in 4 T1 breast cancer model following administration of IL-33. IL-33 promoted tumor growth, development of lung and liver metastases, intratumoral cell proliferation and neovascularisation [18].

While ILC family sub-populations have been investigated in animal models, the role of these cells in protection against human malignancy has been less explored. Nonetheless the potential contribution of these cells has been reviewed extensively [19–23]. Here we examined the frequency of the ILC family in tumour tissues and studied their phenotypic changes.

## Methods

### Patient selection and sample preparation

Samples were obtained from 13 patients with gastrointestinal tumours, 16 patients with malignant breast cancer and 5 patients with benign breast tumours that underwent surgical removal of tumour tissue (Additional file 1: Table S1). Gastrointestinal tumours comprised esophageal, gastric, colon and rectal tumours. Peripheral blood was taken from each patient before commencing anaesthesia. The samples were provided by Xinjiang Tumour hospital and ethical approval was obtained from Xinjiang Tumour Hospital ethical committee and Oxford Tropical Research Ethics Committee (587–16) and London - City & East Research Ethics Committee (16/LO/1607).

Tumor tissue samples were washed repeatedly and then immediately dissociated into single-cell suspensions following surgery using tumour dissociation kit (Miltenyi Biotec 130–095-929). Solution A, Solution B and Solution C from tumor dissociation kit were dissolved by sterile PBS solution and aliquoted into small tubes stored at − 20 degrees. Briefly, samples were cut into small pieces, (1-3 mm) and transferred into C tubes (Miltenyi Biotec 130–096-334). Before loading the C tube into gentleMACS Octo-dissociator (Miltenyi Biotec 130–096-427), 1 unit of Solution A, Solution B and Solution C were added into C tube containing 10 ml RPMI1640. The tubes were then run on ‘h_tumor_01’ and ‘h_tumor_02’ programmes respectively with intervals of 30- min incubation at 37 ͦC with rotation. Following second incubation the tubes were run on ‘h_tumor_3’ programme and samples passed through 70 μm strainer. The cells were centrifuged and washed with RPMI 1640 medium. The enzymatic degradation steps were removed for dissociation of gastrointestinal tumours as this did not render any benefits in terms of cell yield. PBMCs were isolated from whole blood samples using ficoll density gradient centrifugation.

### Flow cytometry

The following anti human antibodies were purchased from Biolegend unless stated otherwise. CD19 (SJ25C1; BD Biosciences), CD11b (DCIS1/18; Abcam), CD11c (BU15), FcεRI (AER-37 (CRA-1)), CD14 (MφP9; BD Biosciences), TCRαβ (IP26), TCRγδ (B1), CD3 (SK7 and OKT3), CD45 (H130), CRTH2 (BM16; Miltenyi Biotec), CD127 (A019D5), c-Kit (104D2), CD44 (G45–26 and IM7), CCR7 (G043H7), PD-1 (EH12.2H7), CTLA-4 (L3D10 and BNI3 BD Biosciences), CD56 (HCD56 and 5.1H1L), CD69 (FN50), KLRG1 (SA231A2), MHC II (L243) and LIVE/DEAD Fixable Violet Dead Cell Stain Kit. Lineage stains were combined through a single channel, which allowed ILC phenotypic marker assessment but limited the potential to correlate with other cell populations.

Samples were acquired using FACSDiva software on LSR Fortessa (Becton Dickson). The flow cytometry data were analysed using FlowJo software.

## Results

### Group 2 ILC are enriched in breast cancer tissue

**Fig. 1:**
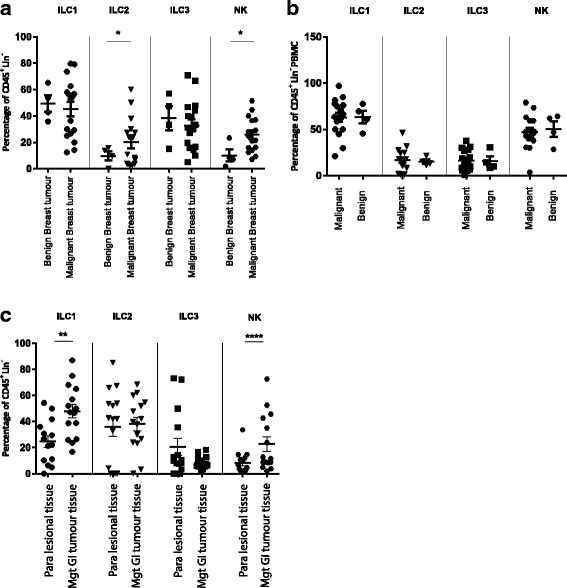
Frequency of ILC populations in breast and GI tumours. Percentage of lineage negative CD45 positive ILC groups in benign (*n* = 4) and malignant breast tumours (*n* = 17) determined by flow cytometry (**a**), PBMCs from patients with benign (*n* = 4) and malignant (*n* = 15) breast tumours (**b**), lesional (*n* = 15) and para-lesional (*n* = 15) gastrointestinal tumour tissues (**c**). Frequency of ILC1, ILC2 and ILC3 is shown as the percentage of lineage negative, CD56^−^, CD45^+^ and NK cells are gated from lineage negative, CD45^+^ cells

To investigate the potential immune surveillance ability of ILC in breast cancer, we compared the frequency of these cells in malignant and benign tissues. To identify ILC, the single cell suspensions of tumours were stained with common lineage markers (CD3, CD14, CD19, CD11c, CD11b, CD56, IL-3R, FcεRI, TCRαβ and TCRγδ), CD45, CRTH2, IL-7Rα and c-Kit. Lineage negative cells expressing haematopoietic and lymphoid markers CD45 and IL-7Rα were selected and based on the expression of CRTH2 and c-Kit were sorted into group 1 ILC (CRTH2^−^ c-Kit^−^), group 2 ILC (CRTH2^+^ c-Kit ^+/−^) and group 3 ILC (CRTH2^−^ c-Kit^+^) (Additional file 1: Figure S1 and Table 1). While the frequency of group 1 and group 3 ILC remained similar, significantly higher percentages (*p* value: 0.04) of ILC2 were observed in malignant cancer tissue compared to benign tissue (Fig. 1a). Furthermore, NK cells were also enriched (*p* value: 0.01) in malignant breast cancer tissue (Fig. 1a). Interestingly we did not observe such differences in the peripheral blood of patients with benign or malignant breast tumours (Fig. 1b). It is important to be cautious about over-interpreting small cell numbers and percentages. We have tried to address this by acquiring all events from each biopsy, and by having large patient cohorts using the same gating strategy throughout.

**Table 1:** ILC populations surface markers

Studied populations	Definition
ILC1	CD45+ Lin- CD56- CD127+ c-Kit- CRTH2-
ILC2	CD45+ Lin- CD56- CD127+ c-Kit−/+ CRTH2+
ILC3	CD45+ Lin- CD56- CD127+ c-Kit+ CRTH2-
NK cells	CD45+ Lin- CD56+

Lineage markers: CD3, CD14, CD19, CD11c, CD11b, CD56, IL-3R, FcεRI, TCRαβ and TCRγδ. ILC were divided into 3 groups based on expression of cell surface markers. ILC1 cells were identified as Lineage negative, CD45^+^, CD127^+^, CD56^−^, CRTH2^−^ and c-Kit^−^. Group 2 ILC were negative for lineage markers, CD56 and expressed CRTH2 and CD127. Group 3 ILC expressed c-Kit, CD45 and CD127, did not express CD56 and CRTH2

### Group 1 ILC and NK cells are enriched in malignant gastrointestinal tissue

In order to investigate whether the differences were unique to breast tissue, we also examined the malignant and para-malignant tissues of different gastrointestinal tumours including eosophageal, gastric, colonic and rectal cancers. Compared to para-tumour tissue, ILC1 (*p* value: 0.001) and NK cells (*p* value: 0.01) were accumulated in the malignant tissues, whereas smaller numbers of ILC3s (*p* value: 0.04) were found in the tumour tissues (Fig. 1c).

### PD-1 and CTLA-4 are upregulated on tumour associated ILC populations

Programmed Death 1 (PD-1, CD279) and Cytotoxic T-Lymphocyte Associated protein (CTLA-4) are inhibitory receptors that modulate immune responses and attenuate tissue damage [24]. Tumour cells can harbor checkpoint receptors to evade immune recognition, and blocking these receptors can augment immune responses against cancer. Interaction of PD-1 with its ligands, PDL1 and PDL2, on macrophages and dendritic cells downregulates IL-2 production, inhibits activation, proliferation and cytokine production [25, 26]. Tumour cells can induce cytotoxic T cell unresponsiveness by acquiring the expression of PD-1 ligands [27]. Monoclonal antibodies targeting PD-1, Lambrolizumab, Nivolumab and Pembrolizumab [28] have favorable immunotherapeutic effects in treatment of a variety of cancers including melanoma, renal and lung cancers [29, 30]. Binding of CTLA-4 to its natural ligands CD80 and CD86 can induce apoptosis and clonal deletion of activated human T cells [31]. CTLA-4 antagonist, Ipilimumab has been approved in treatment of melanoma and breast cancer [32].

**Fig. 2:**
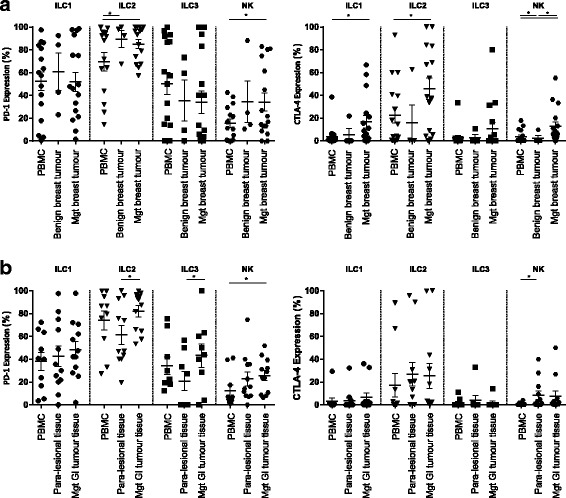
Expression of PD1 and CTLA-4 on ILC populations. Expression of PD1 (**a** left graph) and CTLA-4 (**a** right graph) on ILC groups in peripheral blood of patients with malignant breast tumour (*n* = 14–16), benign breast tumour tissue (*n* = 4) and malignant breast tumour tissue (*n* = 14–16) determined by flow cytometry. Expression of PD1 (**b** left graph) and CTLA-4 (**b** right graph) on ILC groups in peripheral blood of patients with malignant GI tumour (*n* = 8–12), para-lesional tumour tissue (*n* = 8–12) and malignant GI tumour tissue (*n* = 8–12)

To evaluate the presence of immune checkpoints, we examined ILC populations in tumour tissue. Although we did not observe differences in the expression of CTLA-4 and PD-1 on ILC populations in malignant and benign breast tumour tissue (Fig. 2a, and Additional file 1: Figure S2 and Additional file 1: Figure S3), the level of PD-1 was significantly upregulated in tumour associated ILC2 (*p* value: 0.04) and NK cells (*p* value: 0.02) compared to circulating ILC in peripheral blood (Fig. 2a). CTLA-4 was also significantly higher on ILC1 (p value: 0.03), ILC2 (p value: 0.02) and NK cells (p value: 0.005) (Fig. 2a). Interestingly, we observed higher expression of PD-1 on ILC2 (*p* value: 0.03) and ILC3 (p value: 0.04) populations in GI tumours compared to para lesional tissue, while CTLA-4 was not significantly altered (Fig. 2b and Additional file 1: Figure S4 and Additional file 1: Figure. S5).

### High expression of MHC-II, KLRG1, CD69 and CD44 was observed in tumour infiltrating innate lymphoid cells

Successful activation of the immune cells is essential in elimination of tumour cells. To assess the activation status of tumour infiltrating ILC population, we investigated the expression of Major Histocompatibility Complex (MHC)-II, CD69, CD44, Killer cell lectin-like receptor G1 (KLRG1) and CCR7 in peripheral blood, benign and malignant breast tumour tissue and malignant and para-malignant tissue in GI cancers (Additional file 1: Figure S2, Additional file 1: Figure S3, Additional file 1: Figure S4, Additional file 1: Figure S5).

#### MHC-II

**Fig. 3:**
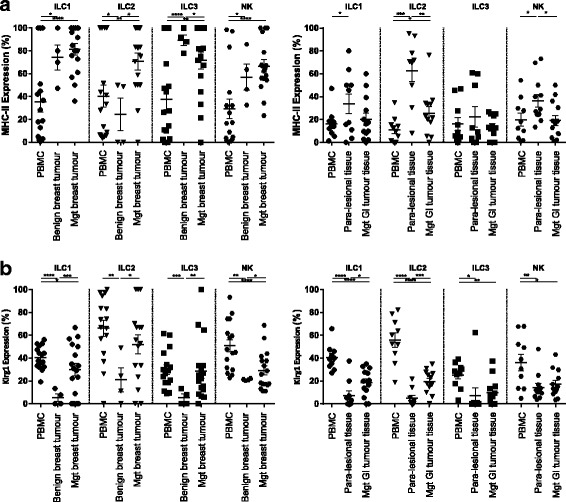
Expression of MHC II and Klrg1 on ILC populations. Expression of MHC II (**a** left graph) and Klrg1 (**b** left graph) on ILC groups in peripheral blood of patients with malignant breast tumour (*n* = 16–17), benign breast tumour tissue (*n* = 4) and malignant breast tumour tissue (*n* = 16–17) determined by flow cytometry. Expression of MHC II (**a** right graph) and Klrg1 (**b** right graph) on ILC groups in peripheral blood of patients with malignant GI tumour (*n* = 9–12), para-lesional tumour tissue (*n* = 9–12) and malignant GI tumour tissue (*n* = 9–12)

Recently it has been shown that innate lymphoid cells can enhance adaptive immune responses and promote inflammation either indirectly by producing cytokines and induction of lymphocyte priming through dendritic cells or directly by MHC-II dependent interactions with T cells [33–35]. ILC populations in benign (*p* values ILC1: 0.01, ILC2: 0.01, ILC3:0.0001, NK: 0.04) and malignant (p values ILC1: 0.0001, ILC2: 0.009, ILC3:0.003, NK: 0.0005) breast tumour tissue express significantly higher level of HLA-DR compared to peripheral blood (Fig. 3a, and Additional file 1: Figure S2 and Additional file 1: Figure S3). Similarly, we observed a significant increase in expression of MHC-II on the ILC2 (p value: 0.02) population in the GI malignant tissue compared to peripheral blood but this difference was not found in ILC1 and ILC3 populations (Fig. 3a, and Additional file 1: Figure S4 and Additional file 1: Figure S5).

#### KLRG1

Both NK and antigen experienced T cells can express co-inhibitory receptor killer-cell lectin like receptor G1 (KLRG1). Around 20%–40% of T cells in young adults can express this marker [36] which rises to 90% in individuals over 65 years of age [37]. On T cells, KLRG1 has been considered a marker of senescence and memory population. Effective immunotherapy in mice with B cell lymphoma enhances expansion of KLRG1 expressing T lymphocytes [38].

ILC populations, in mice and human, express KLRG1 receptor which is an indicator of activation. A population of IL-25 responsive KLRG1^hi^ group 2 innate lymphoid cells, designated inflammatory ILC2s ‘iILC2’ was discovered in mice following infection. These cells showed more plasticity than KLRG1^int^ cells and could give rise to IL-17A producing ILC3s [39]. Higher frequencies of activated KLRG1 expressing ILC2 were detected in skin lesions of atopic dermatitis patients [10]. KLRG1 expressing NK cells protect against pulmonary metastatic disease [40] and colorectal carcinoma [41].

Investigating the expression of KLRG1 on tumour infiltrating ILC populations showed that a higher percentage of ILC in blood (*p* values ILC1: 0.0001, ILC2: 0.005, ILC3: 0.0002, NK: 0.0001) and breast tissue (*p* values ILC1: 0.0006, ILC2: 0.02, ILC3: 0.002, NK: 0.03) of patients with malignant breast tumour express KLRG1 compared to patients with benign tumours, consistent with a more activated phenotype (Fig. 3b, and Additional file 1: Figure S2 and Additional file 1: Figure S3). Similarly, ILC1 (*p* value: 0.01) and ILC2 (p value: 0.0009) populations in malignant GI tumour tissue express significantly elevated KLRG1 compared to paramalignant tissue (Fig. 3b and Additional file 1: Figure S4 and Additional file 1: Figure S5).

#### CD69 and CCR7

The glycoprotein CD69 is a marker of early activation on lymphocytes [42]. Following activation in lymph nodes, lymphocytes upregulate the expression of CD69 which influences sensing the sphingosine 1 phosphate 1 (S1P1) gradient. This gradient directs lymphocytes between tissue and lymph nodes. Therefore, expression of CD69 facilitates the peripheral tissue retention of cells [43].

In contrast, the chemokine receptor CCR7 binds to CCL19 and CCL21 and guides lymphocytes into the lymph nodes [43]. Tumour infiltrating lymphocytes have higher expression of CD69 and lower expression of CCR7.

Although the contribution of CD69 and CCR7 on trafficking of innate lymphoid cells is yet to be established, several studies have shown the up-regulation of CD69 in ILC activation, and CCR7 dependent migration of ILC to draining lymph nodes [44–47].

**Fig. 4:**
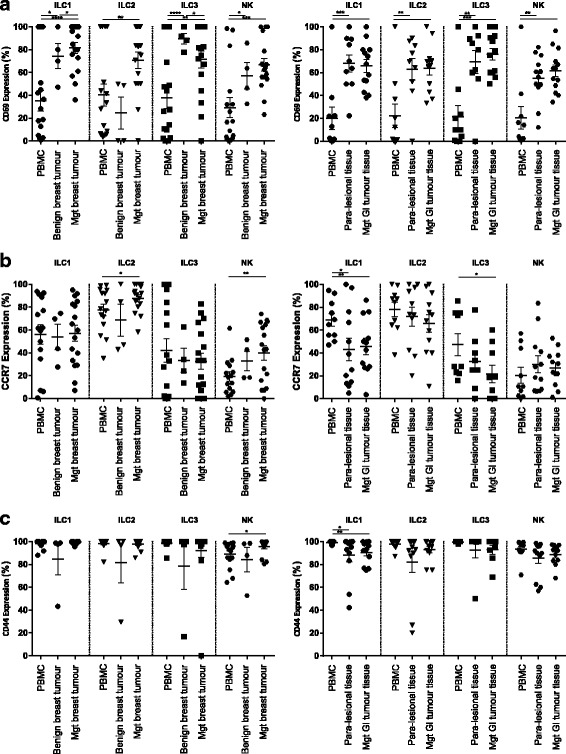
Expression of CD69, CCR7 and CD44 on ILC populations. Expression of CD69 (**a** left graph), CCR7 (**b** left graph) and CD44 (**c** left graph) on ILC groups in peripheral blood of patients with malignant breast tumour (*n* = 14–17), benign breast tumour tissue (*n* = 4) and malignant breast tumour tissue (*n* = 14–17) determined by flow cytometry. Expression of CD69 (**a** right graph), CCR7 (**b** right graph) and CD44 (**c** right graph) on ILC groups in peripheral blood of patients with malignant GI tumour (*n* = 9–13), para-lesional tumour tissue (*n* = 9–13) and malignant GI tumour tissue (*n* = 9–13)

Evaluating the phenotype of tumour infiltrating ILC in breast and malignant GI carcinomas showed a significantly higher frequency of ILC expressing the early activation marker CD69 compared with circulating innate lymphocytes (Fig. 4a and Additional file 1: Figure S2, Additional file 1: Figure S3, Additional file 1: Figure S4, Additional file 1: Figure S5). Furthermore, the expression of CCR7 was significantly lower on ILC1 (p value: 0.008) and ILC3s (p value: 0.02) isolated from GI tumor tissue when compared to blood. We did not observe significant changes in CCR7 expression in breast cancer tissue compared to peripheral blood (Fig. 4b and Additional file 1: Figure S2, Additional file 1: Figure S3, Additional file 1: Figure S4, Additional file 1: Figure S5).

#### CD44

Membrane glycoprotein CD44 is an activation marker that has been extensively studied in malignant and inflammatory conditions. CD44 contributes to the rearrangement of cytoskeleton and initiation of effector responses in lymphocytes [48]. Blocking CD44 on dendritic cells inhibits activation and proliferation of naïve T helper cells [49]. Activated group 2 ILC have been shown to express high levels of CD44 in lungs and to be involved in allergic airway inflammation [50].

Studying the expression of CD44 on tumour infiltrating ILC and peripheral blood confirmed high expression of CD44 on all ILC populations in patients with breast or malignant GI tumours (Fig. 4c and Additional file 1: Figure S2, Additional file 1: Figure S3, Additional file 1: Figure S4, Additional file 1: Figure S5).

## Discussion

Since identification of the innate lymphoid cell family, their emerging roles have included immunity, tissue repair, metabolism and inflammation. A role in tumorigenesis and/or cancer control has been raised but the data are largely based on animal model studies [15, 19, 21]. Understanding of immune regulation of malignancies is important for the development of future therapeutic strategies. Here, albeit with caveat of the challenges of cell numbers obtained when handling human tissue biopsies, we show enrichment of ILC populations within different cancers, together with altered activation status. These findings would support a role for ILC in cancer pathogenesis, however such correlative data do not prove causality.

IFN-γ is the cardinal cytokine produced by ILC1 and it is a critical regulator of anti-tumour responses. The anti-proliferative activity of IFN-γ is mediated by activation of STAT-1 and induction of p21^WAF1/CIP1^ and p27^Kip1^ that bind to cyclin dependent kinases-2 and 4 (CDK) [51]. IFN-γ/STAT1 pathway also induces caspase 1, FAS and FASL and promotes apoptosis [52]. Production of IFN-γ by NKT cells enhances cytolytic activity of NK effector cells [53]. The anti-tumour activity of ILC1 like cells were shown in a PyMT mouse mammary tumor model. These cells express high levels of NK1.1, CD49a, CD103, granzyme B and TRAIL. ILC1 exhibit potent cytotoxicity against mammary tumours and inhibit cancer growth [15]. Here we observed enrichment of NK and ILC1 cells in malignant gastrointestinal tumours compared to the adjacent tissue.

On the contrary to ILC1 and NK cells, cytokines that activate group 2 ILC may be more protumourigenic. For example, IL-33 promotes chronic inflammation and tumour development in some models. Serum levels of IL-33 are increased in breast, lung, gastric and hepatocellular carcinomas. Blocking IL-33 receptor (ST2) reduced generation and size of tumours in a colon cancer model [54]. In addition, type 2 cytokines are shown to promote generation of tumours. IL-13 can induce progression of prostate tumours [55] and enhance M2 phenotype polarization of macrophages [56]. In patients with gastric cancer, a higher frequency of ILC2 was observed in peripheral blood and was thought to contribute to an immunosuppressive microenvironment [17]. Furthermore, an increase in endogenous levels of IL-33 were observed in the 4 T1 model of breast cancer that correlated with cancer progression and metastasis. ILC2, alternatively activated M2 macrophages and myeloid derived suppressor cells accumulate in intramural tissue following administration of IL-33 [18]. Similar to observations in the 4 T1 mouse model of breast cancer, we detected higher frequency of activated ILC2 in human malignant breast tumour tissue compared to benign tissue.

Both pro- and anti- tumour properties of group 3 innate lymphoid cells have been observed. 129SvEv.RAG^−/−^ mice infected with H.*hepaticus* and treated with the carcinogen azoxymethane (AOM) develop IL-22 dependent colorectal cancer. Colonic ILC3s are thought to be the source of IL-17A and IL-22 and are found in higher frequencies in this model [16]. In contrast, in human non-small cell lung cancer (NSCLC) NKp44+ ILC3 accumulate in tertiary lymphoid structures. These cells are a source of IL-22, TNF-α, IL-8 and IL-2. ILC3 were found in higher frequencies in early stages of tumour and their presence correlated with density of protective lymphoid structures inside the tumour [57].

Comparing tumour infiltrating ILC populations with circulating ILC, showed that ILC populations have an activated phenotype in tumour tissue with higher expression of MHC-II, KLRG1, CD69 and CD44. Unlike adaptive immune responses, ILC populations lack RAG-dependent rearranged antigen receptors. They can be activated by direct interactions mediated by pattern recognition receptors (PRR) and natural cytotoxicity receptors (NCR) or indirectly by activating cytokines. Tumour associated ILC are an early source of innate cytokines. Direct interaction with cancer cells and inflammatory tumour microenviroments may contribute to their activated phenotype. Here we present an observational study, but it will clearly be important to proceed to define the underlying mechanistic associations.

Functional diversity of ILC populations combined with expression of similar activatory/ inhibitory receptors make it difficult to extrapolate the use of blocking or activatory agents. For instance blocking inhibitory receptors KLRG1, PD1 and CTLA-4 on ILC1 population may help in increasing production of anti-tumour cytokines, IFN-γ and TNF-α, but as these receptors are also expressed highly on ILC2 population in tumour environments, using blocking agents might contribute to increase in type 2 cytokine production and modulate tumour growth. Further studies are crucial to find strategies to specifically target ILC subpopulations to define underlying mechanisms and new approaches to treatment.

Most studies on the role of innate lymphoid cells in cancer have been done on animal models. Therefore, further clinical investigations in humans are required to define the extent of their role in cancer. It is likely that not only the intrinsic ability of ILC but also the nature of transformed cells and tumour microenvironment are essential determinant factors. Further understanding of their role provides novel targets in immunotherapeutic strategies to treat cancer.

## Conclusions

Innate lymphoid cells infiltrate tumours and show altered activation status. These findings would be consistent with a role for innate lymphoid cells in disease or its control. Further understanding of mechanism may contribute to future therapeutic advance.

## Additional file

Additional file 1: **Figure S1.** Representative staining of ILC populations. ILC were gated on lineage negative, CD45+, CD127+ cells and based on the expression of CRTH2 and c-Kit divided into ILC1 (CRTH2-, c-Kit-), ILC2 (CRTH2+, c-Kit +/−) and ILC3 (CRTH2-, c-Kit+). **Figure S2** Representative example of ILC1, ILC2, ILC3 phenotypic analysis from benign breast tissue. **Figure S3** Representative example of ILC1, ILC2, ILC3 phenotypic analysis from malignant breast tissue. **Figure S4** Representative example of ILC1, ILC2, ILC3 phenotypic analysis from malignant GI tumour tissue. **Figure S5** Representative example of ILC1, ILC2, ILC3 phenotypic analysis from paralesional GI tumour tissue. **Table S1** Patient characteristics. (PDF 5287 kb)

## Abbreviations

CTLA-4: Cytotoxic T-lymphocyte-associated protein 4
IFN: Interferon
ILC: Innate lymphoid cells
KLRG-1: Killer cell lectin-like receptor 1
MHC: Major histocompatibility complex
NCR: Natural cytotoxicity receptor
NK: Natural Killer
PD-1: Programmed cell death protein 1
PDL1: Programmed death-ligand 1
