# Supplementary material for: Activated innate lymphoid cell populations accumulate in human tumour tissues
Source: BMC Cancer. 2018 Mar 27;18:341. doi: 10.1186/s12885-018-4262-4 (PMC5870240; doi:10.1186/s12885-018-4262-4)
Supplement: Supplementary file 1 — Figure S1. Representative staining of ILC populations. ILC were gated on lineage negative, CD45+, CD127+ cells and based on the expression of CRTH2 and c-Kit divided into ILC1 (CRTH2-, c-Kit-), ILC2 (CRTH2+, c-Kit +/−) and ILC3 (CRTH2-, c-Kit+). Figure S2 Representative example of ILC1, ILC2, ILC3 phenotypic analysis from benign breast tissue. Figure S3 Representative example of ILC1, ILC2, ILC3 phenotypic analysis from malignant breast tissue. Figure S4 Representative example of ILC1, ILC2, ILC3 phenotypic analysis from malignant GI tumour tissue. Figure S5 Representative example of ILC1, ILC2, ILC3 phenotypic analysis from paralesional GI tumour tissue. Table S1 Patient characteristics. (PDF 5287 kb) [file 12885_2018_4262_MOESM1_ESM.pdf]

Supplemental Figure 1

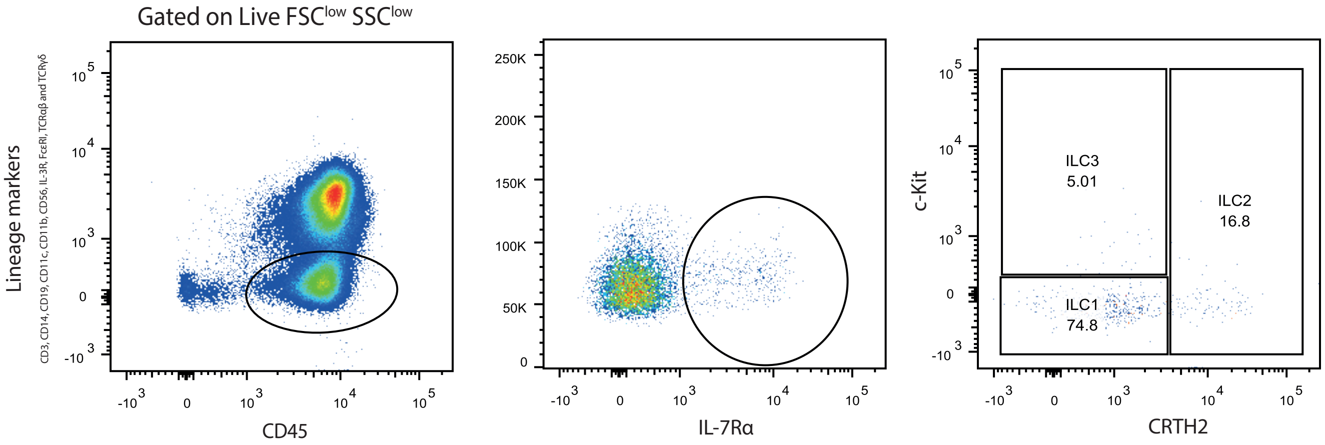

Representative staining of ILC populations. ILC were gated on lineage negative, CD45+ , CD127+ cells and based on the expression of CRTH2 and c-Kit divided into ILC1 (CRTH2-, c-Kit-), ILC2 (CRTH2+, c-Kit +/-) and ILC3 (CRTH2-, c-Kit+).

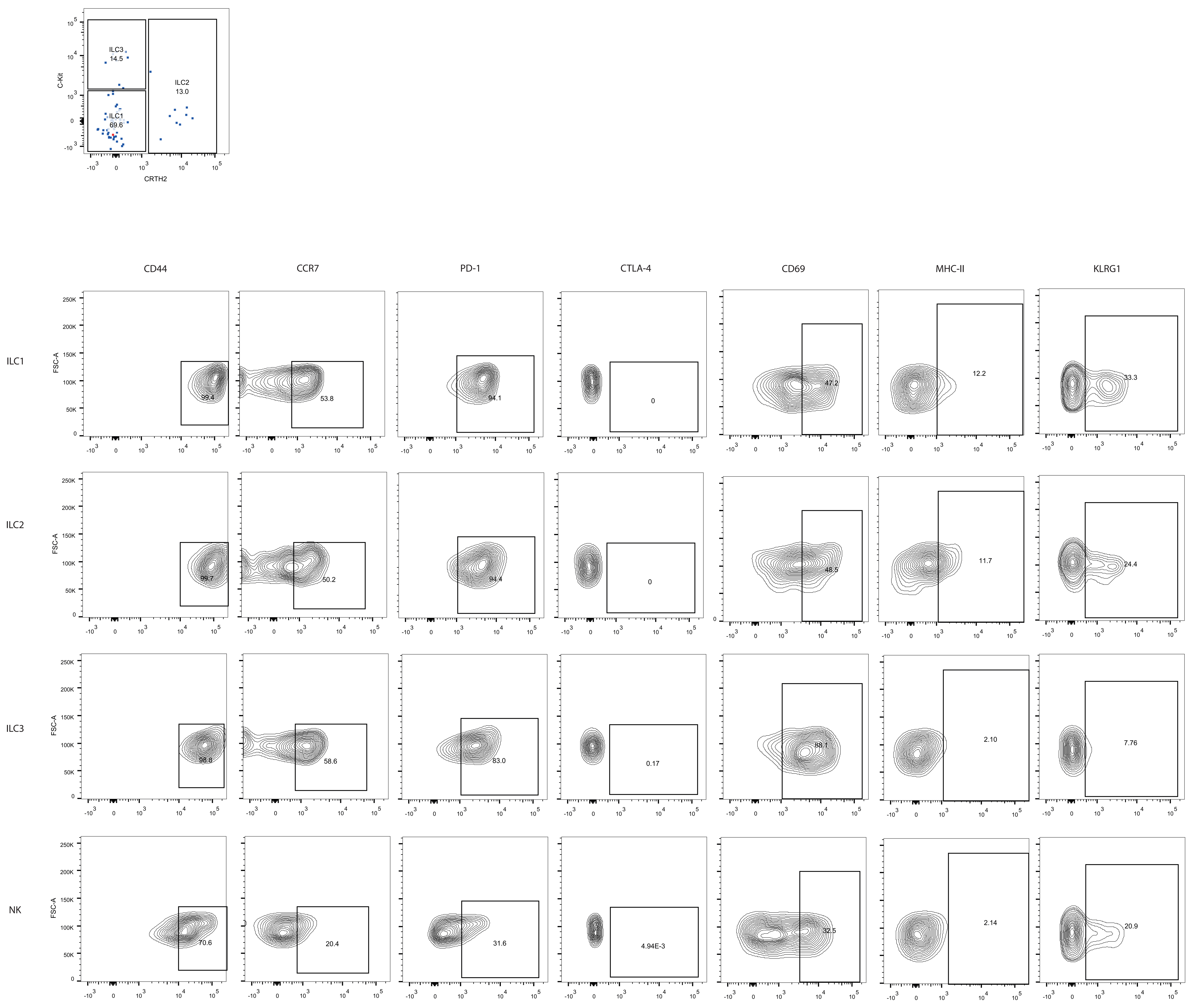

Supplemental Figure 2. Representative example of ILC1, ILC2, ILC3 phenotypic analysis from benign breast tissue.

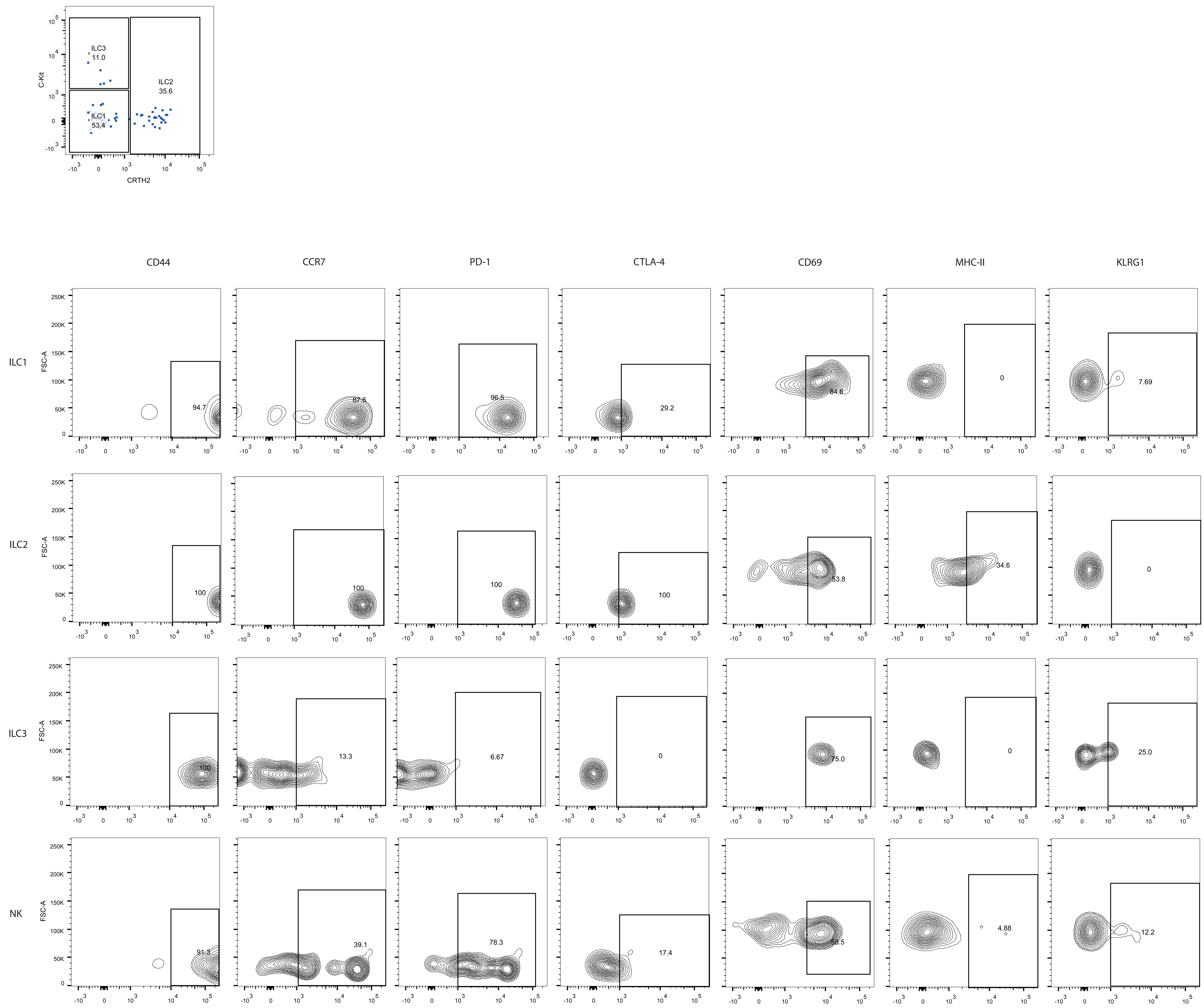

Supplemental Figure 3. Representative example of ILC1, ILC2, ILC3 phenotypic analysis from malignant breast tissue.

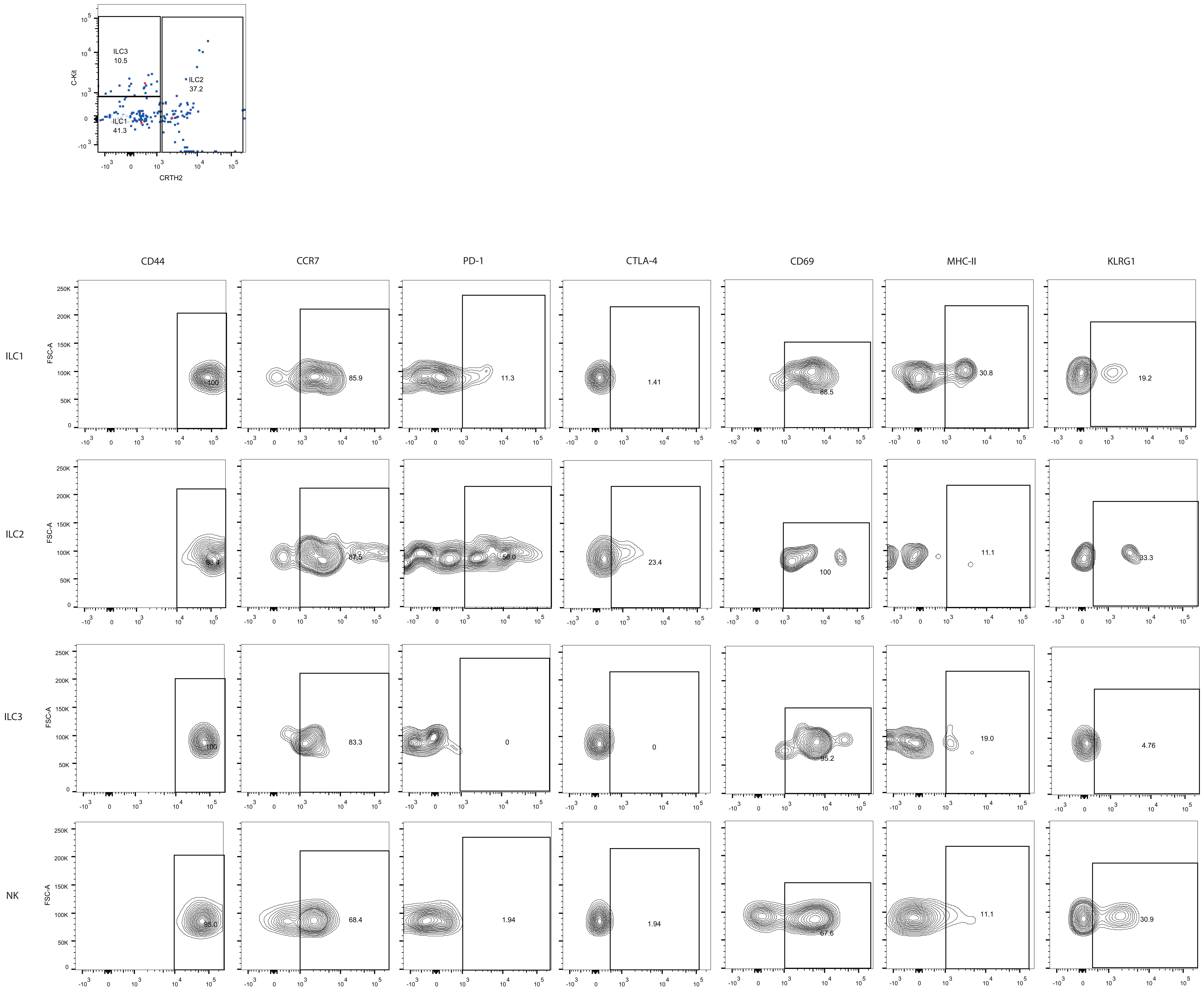

Supplemental Figure 4. Representative example of ILC1, ILC2, ILC3 phenotypic analysis from malignant GI tumour tissue.

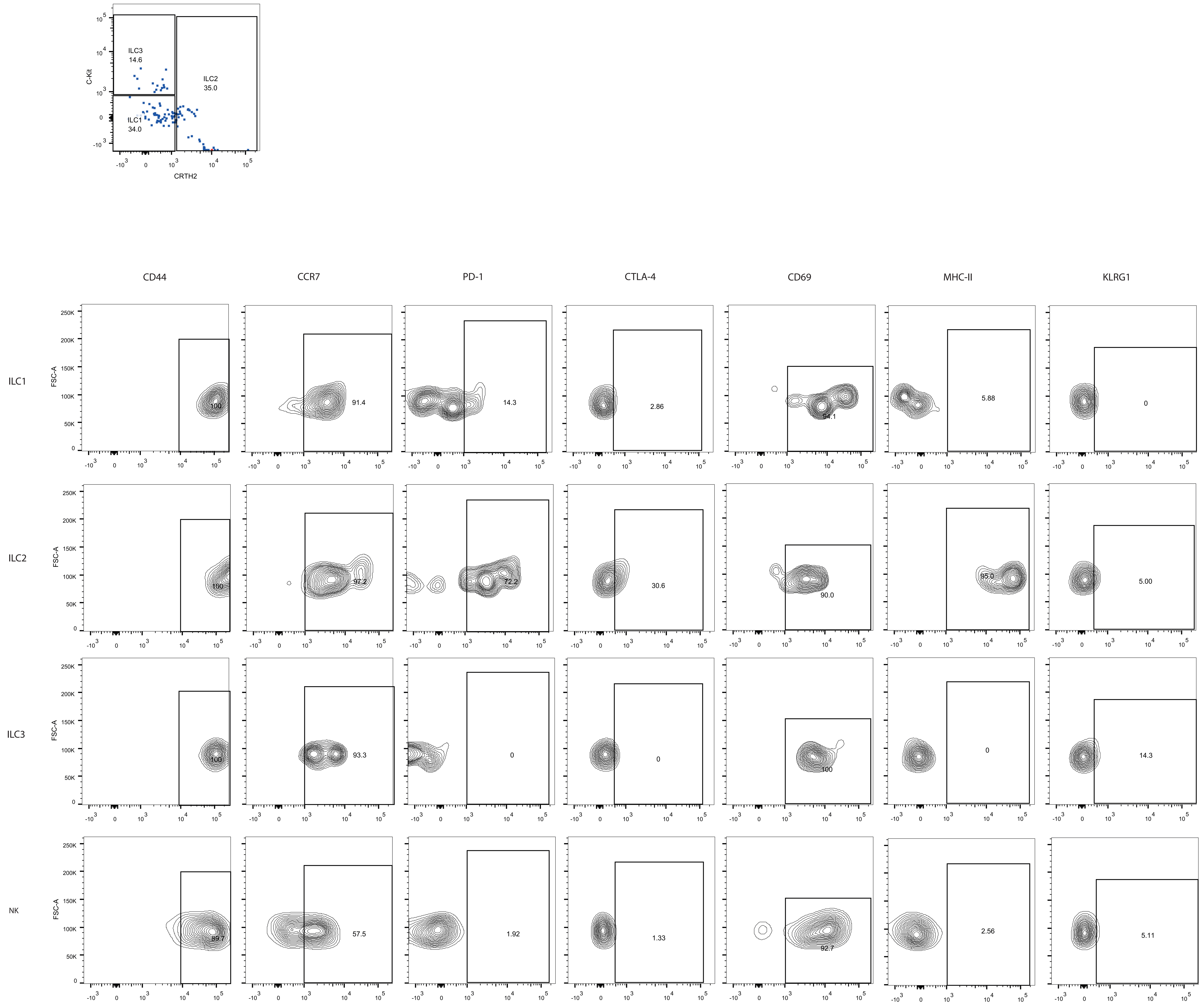

Supplemental Figure 5. Representative example of ILC1, ILC2, ILC3 phenotypic analysis from paralesional GI tumour tissue.

Table S1, patient characteristics

ID	Age	Diagnosis
1	40	bilateral breast fibroadenoma
2	48	left breast malignant tumor (invasive ductal carcinoma)
3	63	right breast invasive ductal carcinoma
4	71	esophagogastric junction malignant tumor
5	42	esophagogastric junction adenocarcinoma
6	54	left breast malignant tumor (invasive ductal carcinoma)
7	22	bilateral breast fibroadenoma
8	42	right breast invasive ductal carcinoma
9	42	right breast benign tumor (inflamatory lesion)
10	47	breast malignant tumor(invasive ductal carcinoma)
11	44	breast invasive ductal carcinoma
12	50	breast invasive lobular carcinoma and lobular carcinoma in situ
13	50	left breast invasive ductal carcinoma
14	44	right breast malignant tumor(invasive Basal-Like)

15	61	sigmoid colon adenocarcinoma
16	74	gastric antrum malignant adenocarcinoma
17	55	rectal malignant carcinoma
18	44	breast ductal carcinoma in situ
19	79	ascending colon adenocarcinoma
20	53	breast invasive ductal carcinoma
21	41	breast tumor (invasive ductal carcinoma)
22	65	left breast invasive ductal carcinoma
23	74	rectal carcinoma
24	47	rectal carcinoma
25	47	stomach malignant tumor (ulcerative adenocarcinoma of greater curvature of stomach)
26	67	left breast invasive ductal carcinoma
27	43	right breast mastitis
28	69	rectum carcinoma
29	86	cardiac tumor carcinoma
30	66	transverse colon malignant carcinoma
31	57	rectal malignant tumor carcinoma

32	41	left breast invasive ductal carcinoma
33	45	left breast fibroadenoma
34	44	left breast invasive ductal carcinoma
